# Editorial: Synergistic effects of PDT and RT in cancer treatment: innovations and challenges

**DOI:** 10.3389/fonc.2026.1810750

**Published:** 2026-03-12

**Authors:** Kave Moloudi, Siamak Haghdoost

**Affiliations:** 1 Laboratoire Aliments, Bioprocédés, Toxicologie Environnements (ABTE, UR4651), University of Caen, Caen, France; 2 Advanced Resource Center for HADrontherapy in Europe (ARCHADE), Caen, France

**Keywords:** laser, photodynamic therapy, photosensitizer agent, radiotherapy, ROS

Photodynamic therapy (PDT) and radiotherapy (RT) are established cancer treatment modalities that exert cytotoxic effects primarily through the generation of reactive oxygen species (ROS) and oxidative stress. Despite their individual clinical success, both approaches have intrinsic limitations, including restricted light penetration and oxygen dependence in PDT, and dose-limiting toxicity and radioresistance in RT. Growing evidence suggests that combining PDT and RT may produce synergistic antitumor effects by amplifying ROS production, enhancing DNA damage, and modulating the tumor microenvironment. This editorial summarizes recent advances in PDT–RT combination strategies, including radiodynamic therapy, nanotechnology-based co-delivery systems, and scintillating nanoparticles capable of converting X-rays into visible light for deep-tissue PDT activation. We discuss mechanistic insights into oxidative stress amplification, immune activation, and immunogenic cell death, as well as strategies to overcome tumor hypoxia and resistance. Additionally, we highlight emerging preclinical and early clinical findings that support improved therapeutic efficacy and potential dose reduction. Despite promising results, significant challenges remain, including optimization of treatment sequencing, standardization of dosing protocols, and validation through large-scale clinical trials. Continued interdisciplinary research is essential to translate PDT–RT synergy into safe and effective clinical applications.

Cancer remains a major global health challenge, necessitating continuous innovation in therapeutic strategies that enhance tumor control while minimizing toxicity and preserving patient quality of life. Radiotherapy (RT) remains a cornerstone of cancer treatment, whereas Photodynamic Therapy (PDT) has emerged as a selective, minimally invasive modality with established efficacy in several malignancies. Despite their individual success, both approaches possess intrinsic limitations. However, *“Synergistic Effects of PDT and RT in Cancer Treatment: Innovations and Challenges”* highlights the growing scientific momentum toward integrating these two oxidative stresses–based modalities to improve therapeutic outcomes.

PDT relies on light activation of a photosensitizer in the presence of molecular oxygen, generating reactive oxygen species (ROS) such as singlet oxygen that induce tumor cell death through oxidative stress, vascular damage, and immune activation. However, limited light penetration depth restricts PDT applications primarily to superficial or accessible tumors. Conversely, RT uses ionizing radiation to induce DNA damage and free radical formation in deep-seated tumors, but its effectiveness is reduced in hypoxic microenvironments and constrained by dose-dependent toxicity to surrounding normal tissues (1, 2).

The rationale for combining PDT and RT arises from their complementary ROS-mediated mechanisms. PDT induces localized oxidative stress, whereas RT produces widespread ionization events, and DNA single- and double-strand breaks. When applied together, these modalities may exceed tumor antioxidant capacity, amplify cytotoxicity, and potentially allow radiation dose reduction without compromising efficacy (3). Such synergy could improve the therapeutic ratio, a central objective in oncology.

Emerging experimental evidence supports this hypothesis. Takahashi et al. demonstrated that 5-aminolevulinic acid (5-ALA)–based radiodynamic therapy combined with single-dose X-ray irradiation significantly suppressed tumor growth in a colorectal cancer xenograft model. The combination enhanced ROS-mediated tumor destruction and increased immune cell proximity to malignant cells without significant systemic toxicity. These findings suggest that tumor-selective photosensitizer accumulation can potentiate RT effects and promote immunogenic cell death.

Clinical insights further illustrate translational potential. A case series by Alves et al. reported the use of antimicrobial PDT (aPDT) to manage severe acute radiodermatitis in patients undergoing head and neck radiotherapy. Rapid improvement in skin lesions and reduction in severity scores demonstrated that PDT may mitigate RT-associated toxicity, thereby improving treatment tolerability. This supportive-care application broadens the conceptual framework of PDT–RT synergy beyond direct tumor cytotoxicity.

Additionally, Li et al. described a case in which PDT combined with Chinese herbal medicine achieved tumor reduction and symptomatic improvement in advanced hypopharyngeal carcinoma. Although anecdotal, this report underscores the potential integration of PDT within multimodal and supportive oncologic care strategies aimed at enhancing patient adherence and overall outcomes.

Mechanistically, the synergy between PDT and RT extends beyond direct oxidative injury. Both modalities can induce immunogenic cell death characterized by the release of damage-associated molecular patterns (DAMPs) and tumor antigens, promoting dendritic cell activation and adaptive immune responses. Combined therapy may therefore exert dual effects: immediate tumor cytotoxicity and longer-term immune-mediated control (3). Such findings align with the broader trend of integrating oxidative therapies with immunotherapy to potentiate systemic antitumor immunity.

Technological advances are accelerating progress in this field. Nanotechnology-based systems capable of co-delivering photosensitizers and radiosensitizers offer improved tumor targeting and ROS amplification (3). Scintillating nanoparticles that convert X-rays into visible light provide a particularly innovative solution to the classical limitation of PDT light penetration, enabling *in situ* activation of photosensitizers during RT. Precision RT techniques, including image-guided radiotherapy, further enhance spatial coordination of combined modalities, optimizing tumor targeting while sparing healthy tissue.

Despite promising developments, challenges remain. Tumor hypoxia limits ROS generation for both PDT and RT, necessitating strategies to enhance oxygenation or optimize treatment sequencing. Standardization of dosing protocols, timing intervals, and patient selection criteria is still lacking. Most available data derive from preclinical models or limited case reports; therefore, well-designed clinical trials are urgently needed to establish safety, efficacy, and long-term outcomes.

In conclusion, the integration of PDT and RT represents a promising frontier in cancer treatment. By leveraging complementary ROS-mediated mechanisms, enhancing immune activation, and incorporating nanotechnology innovations, PDT–RT combinations hold potential to improve therapeutic outcomes while reducing toxicity. Continued interdisciplinary research and robust clinical evaluation will be essential to translate these advances into routine oncologic practice. Combination therapy using PDT and RT demonstrates synergistic effects, achieving higher therapeutic efficacy and lower side effects compared to either treatment alone. Clinical trials and preclinical studies report enhanced tumor response, reduced recurrence, and promising outcomes across multiple cancer types, including lung, breast, cholangiocarcinoma, and cervical cancers.

Despite these encouraging results, critical parameters remain unresolved, such as the optimal light and photosensitizer doses, radiation dose, timing between treatments, and potential complication risks. Addressing these gaps is essential for safe and effective clinical application.

Future directions focus on innovative strategies to improve treatment efficacy, including the use of intracellular light-emitting nanoparticles to overcome limited light penetration and the development of personalized protocols to maximize synergistic benefits while minimizing toxicity.
